# Supplementary figures and images for: Prediction of virus-host associations using protein language models and multiple instance learning
Source: PLoS Comput Biol. 2024 Nov 19;20(11):e1012597. doi: 10.1371/journal.pcbi.1012597 (PMC11614202; doi:10.1371/journal.pcbi.1012597)

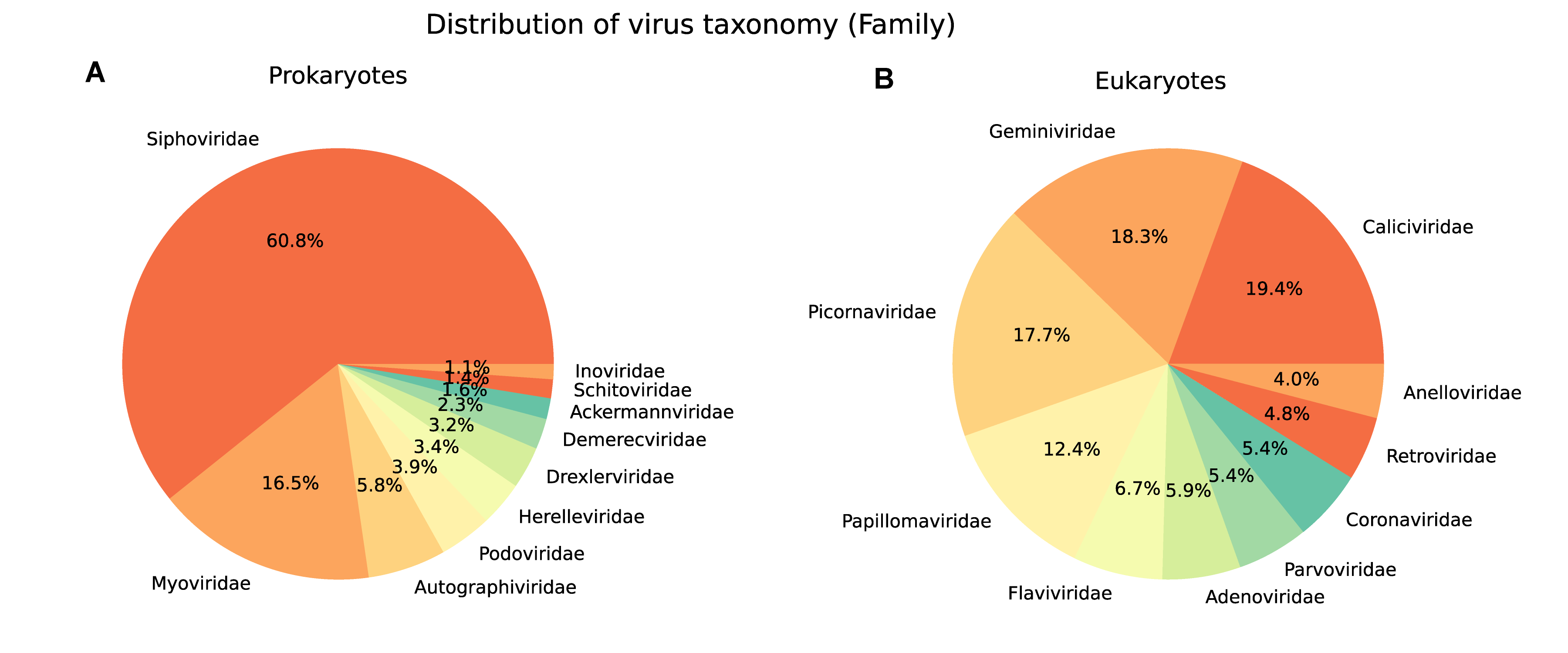

Supplement: S1 Fig — To illustrate the virus taxonomy distribution, we plot pie charts to show the distribution of virus families in prokaryotic and eukaryotic hosts. Viruses associated with prokaryotes are dominated by Siphoviridae family, which constitutes approximately 60% (A), whereas the Geminiviridae, Picornaviridae and Papillomaviridae families are the top three ranked families, each accounting for roughly 18% (B). (TIF) [file pcbi.1012597.s009.tif]

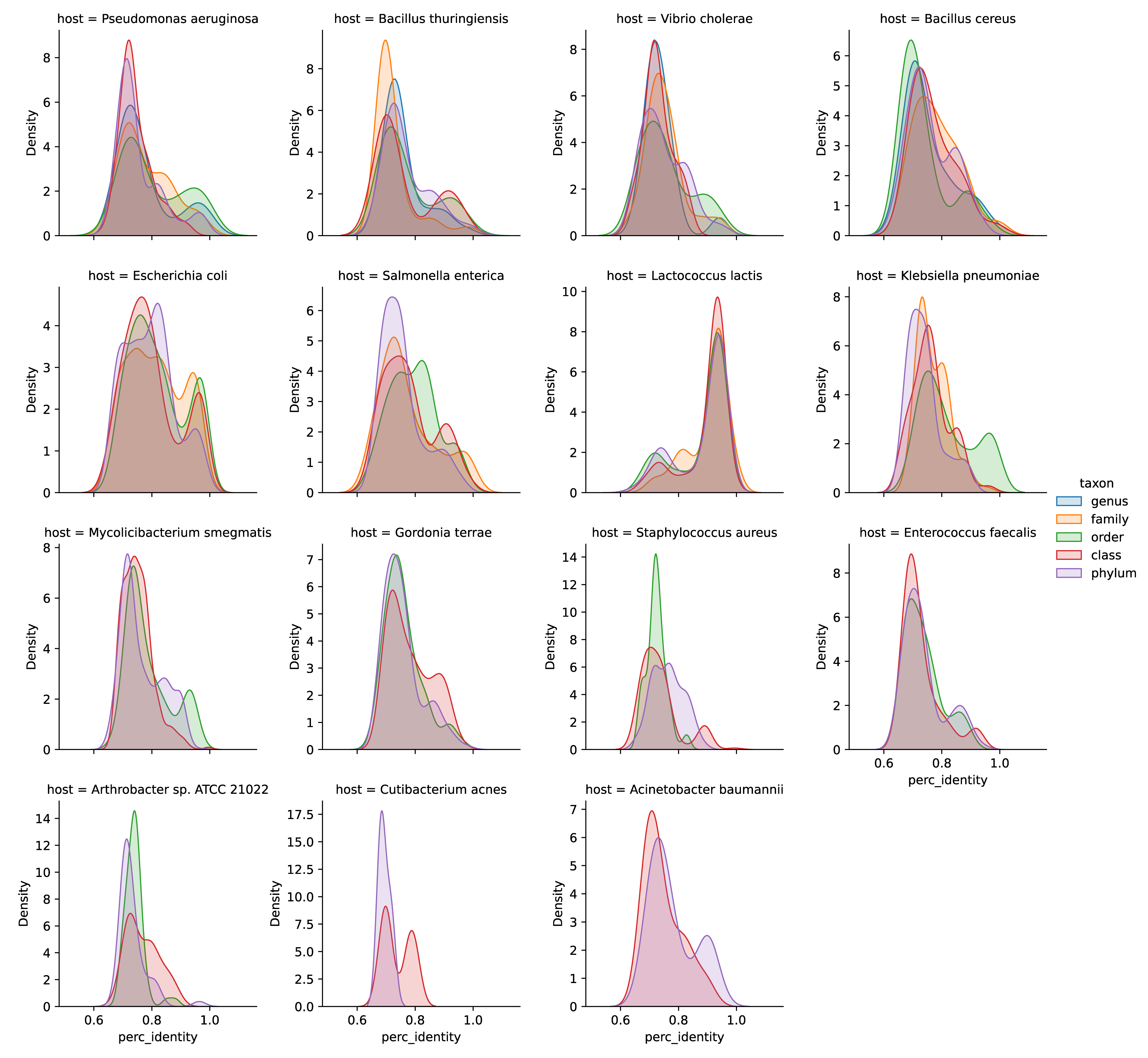

Supplement: S2 Fig — This figure presents the distribution of genome similarities between positive and negative samples on each prokaryotic host, where the negative viruses are chosen based on the same taxonomy (genus, family, order, class, phylum) as the positive viruses. (TIF) [file pcbi.1012597.s010.tif]

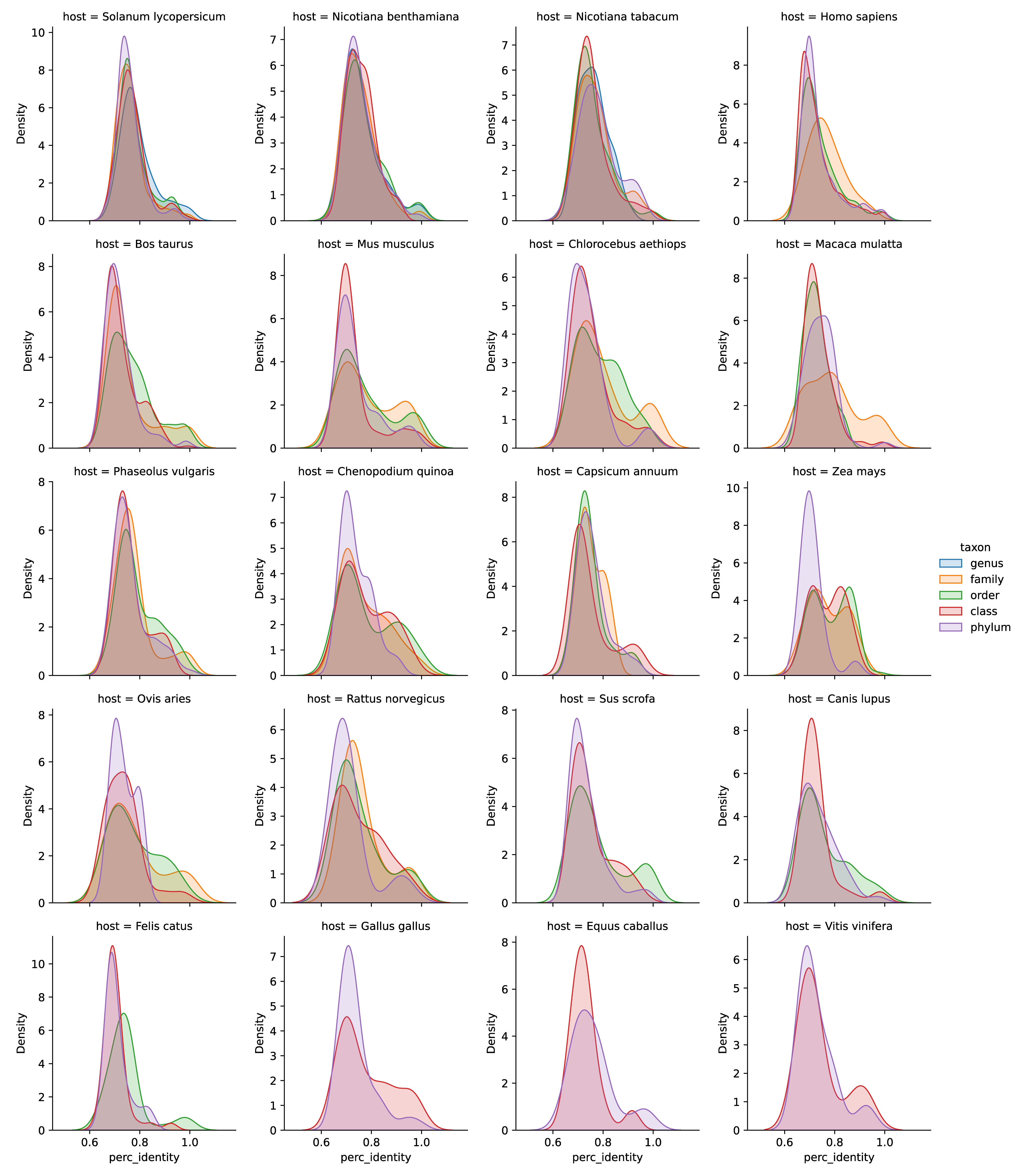

Supplement: S3 Fig — This figure presents the distribution of genome similarities between positive and negative samples on each eukaryotic host, where the negative viruses are chosen based on the same taxonomy (genus, family, order, class, phylum) as the positive viruses. (TIF) [file pcbi.1012597.s011.tif]

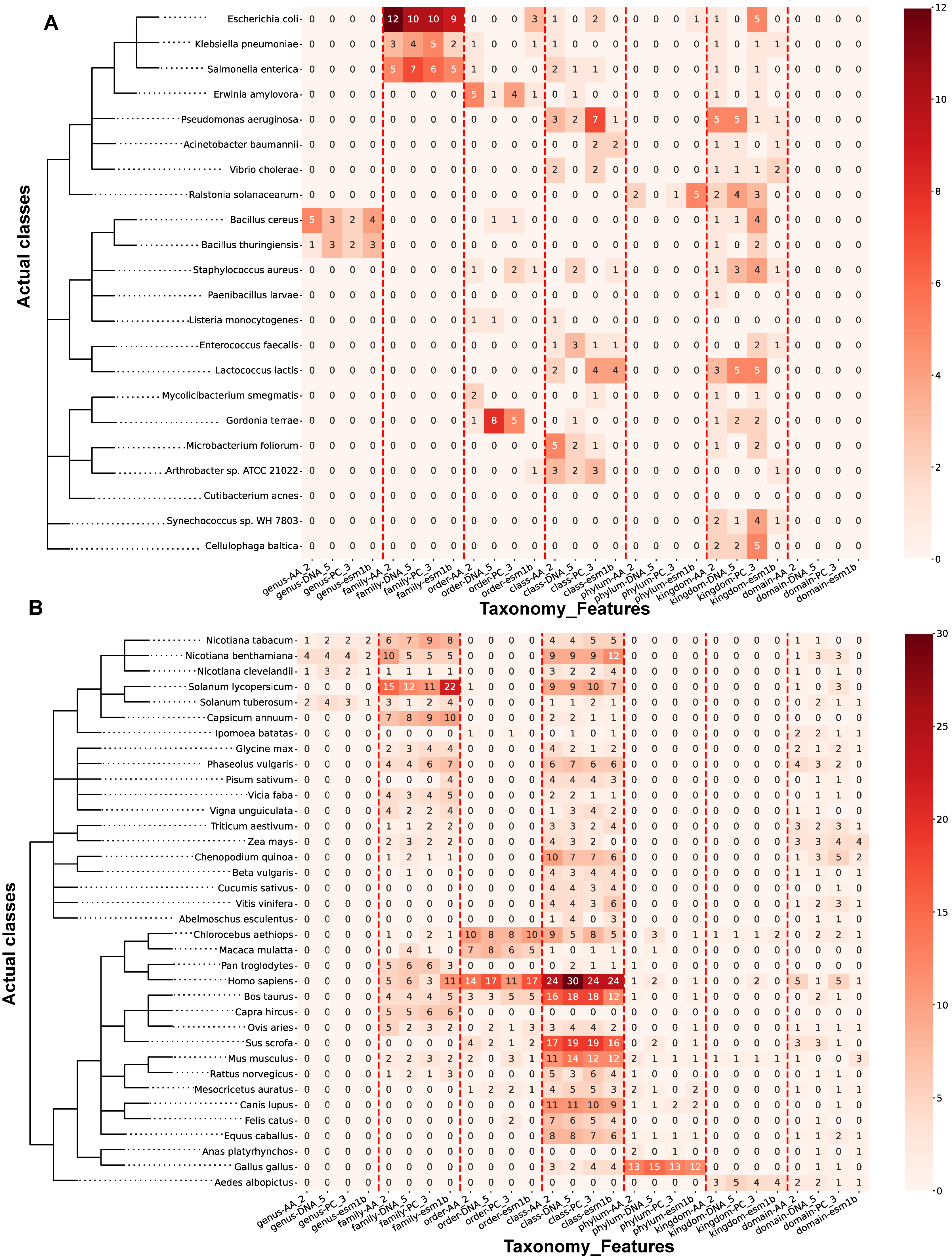

Supplement: S5 Fig — The values in the heatmap are the total number of predicted hosts which belong to the same taxonomy as the true host. (TIF) [file pcbi.1012597.s013.tif]

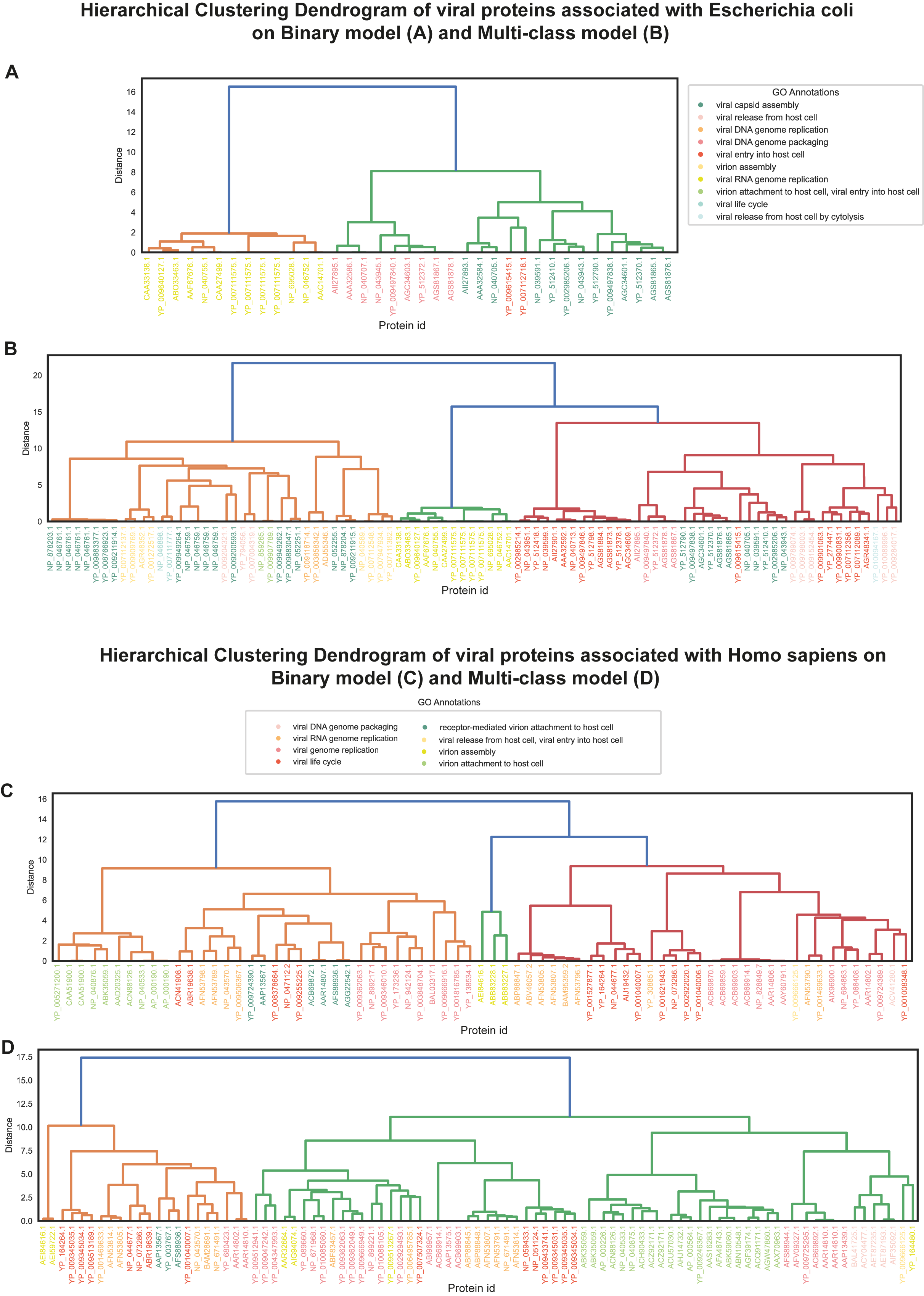

Supplement: S6 Fig — Figures show the hierarchical clustering dendrograms of the top 5 ranked protein embeddings for each virus associated with E. coli (A, B) and H. sapiens (C, D). Here, the protein weights in A and C are obtained by binary models, whereas in B and D, the weights are obtained by multi-class classification models. Protein GO terms regarding the viral life cycle of viruses are highlighted with different colours, allowing us to understand the predictive signals of proteins captured by the pre-trained transformer model. (TIF) [file pcbi.1012597.s014.tif]
