# Supplementary material for: Prediction of virus-host associations using protein language models and multiple instance learning
Source: PLoS Comput Biol. 2024 Nov 19;20(11):e1012597. doi: 10.1371/journal.pcbi.1012597 (PMC11614202; doi:10.1371/journal.pcbi.1012597)
Supplement: Attachment — Submitted filename: point-to-point-reply.pdf [file pcbi.1012597.s015.pdf]

Response to reviewers

We thank the reviewers for their extensive reviews of our manuscript. We believe the revised manuscript has been considerably enhanced by this feedback. We have carefully considered each comment and incorporated the resulting changes into the manuscript, with detailed responses given below. We highlighted the revised

text in the main paper. We welcome any further feedback that the reviewers may be able to offer on our work and thank them again for their time and consideration.

Point to point response:

Reviewer #1: The authors propose a method named EvoMIL for both prokaryotic and eukaryotic virus host prediction. By integrating a pre-trained protein embedding module and a well-designed weighing module (attention-based multiple instance learning), EvoMIL is expected to leverage different proteins in the virus genomes. The idea of the EvoMIL is interesting and the results show that EvoMIL performs good on the dataset created by the author. However, there are still some major concerns about the work, especially the experimental design.

Major concerns:

1. Virus host prediction is a hot research topic, and numerous tools have been developed to address this problem. However, this manuscript lacks benchmark experiments that demonstrate the competitiveness of EvoMIL in comparison to state-of-the-art methods. As a newly designed method, the author should cite these existing tools and provide a comprehensive comparison. A review of many tools was provided here: https://doi.org/10.1093/bioinformatics/btac239 Some of these tools probably have better/newer versions. Although these only focus on prokaryotic viruses, the authors can still compare the performance of this part.

We thank the reviewer for their suggestion and have added benchmarking experiments using some state-of-the-art species-based virus-host predictors. The benchmarking dataset was constructed from the recent iPHoP paper[1]. Compared to our dataset it contains an additional 364 viruses across the 22 prokaryote species that matched the host labels in our training set. We compared EvoMIL prediction performance with 9 methods including the state-of-the-art iPHoP approach. EvoMIL outperforms all of these

baselines, achieving the highest accuracy of 75.27% on the test viruses, with 274 correct predictions out of 364 viruses, with a 33.65% improvement over iPHoP (see section 2.4 “Benchmarking of EvoMIL”). The following figure summarising these results is included in the revised manuscript.

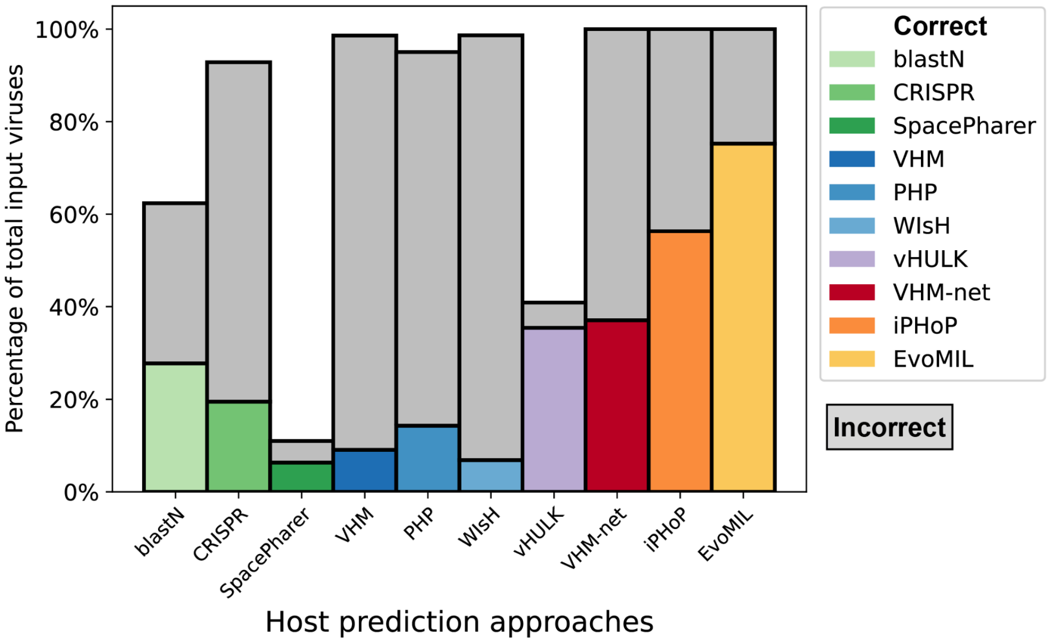

Fig 6. Comparison of EvoMIL and other host prediction approaches on an independent test dataset. The y-axis presents the number of correct predictions (coloured bar) and the number of incorrect predictions (gray bar) for each tool (x-axis) on the chosen benchmarking test dataset (Table S6). This plot shows the percentage of correct and incorrect host species predictions on the test dataset, and prediction source results are available in Table S7.

We have added the following text to our paper:

“Here we compare EvoMIL's predictiton performance with 9 prokaryotic host predictors including the state-of-the-art iPHoP, BLAST, CRISPR, WisH, VirHostMatcher, PHP, SpacePHARER, VirHostMatcher-Net and vHULK. We chose 364 viruses across the 22 prokaryote species that matched the host labels in our training set, which were chosen from the benchmarking test dataset from the iPHoP paper. The full list of the testing viruses can be found in Table S6. For the details about prediction settings for each approach, please see Methods section 4.6.

BLASTn, CRISPR, PHP, WisH, VirHostMatcher and iPHoP make multiple host predictions for a given virus. We considered any predicted host species in their

prediction list that matched the true host species as a correct prediction. EvoMIL, SpacePharer, VHM-Net and vHULK are evaluated based on their top-1 prediction accuracies. As shown in Figure 6, EvoMIL achieves the highest accuracy of 75.27% on the test viruses, with 274 correct predictions out of 364 viruses and has a 33.65% improvement over the state-of-the-art approach, iPHoP."

Reference

[1] Roux S, Camargo AP, Coutinho FH, Dabdoub SM, Dutilh BE, Nayfach S, Tritt A. iPHoP: An integrated machine learning framework to maximize host prediction for metagenome-derived viruses of archaea and bacteria. PLoS biology. 2023 Apr 21;21(4):e3002083.

2. In addition to the existing tools, the authors also discuss the potential use of alignment-based methods for host prediction. Considering that EvoMIL aims to assess the significance of viral proteins in host prediction**,** I suggest that the authors include BLASTp results as a fundamental benchmark method in their experiments. For instance, in the binary classification task, it would be valuable to examine whether BLASTp can yield better alignment results for positive pairs compared to negative pairs. This would provide insights into the performance of EvoMIL.

Thank you for your suggestion. Using BLASTp for protein-based virus-host prediction is an intriguing idea, however, to the best of our knowledge, there isn't an existing published implementation. The challenge is how to rank the host from the multiple hits of the multiple virus's proteins. For example, with the prokaryote datasets, each phage has around 100 proteins, the results of BLASTp will give multiple lists of potential hits. The problem is how to combine the multiple scores to rank the potential host. This would require optimising and validating a scoring method, therefore substantial method development work and out of the scope of our paper. We have instead benchmarked against iPHoP which incorporates features from BLASTp in an ensemble classifier.

3. Since novel viruses are typically reconstructed from metagenomic data, it is possible that the viral sequences obtained may not be as complete as those downloaded from curated databases. Consequently, certain proteins may be missing from the assembled viral contigs. As EvoMIL is a "protein-based" method, I am wondering whether it can still perform effectively on short contigs.

Predicting the hosts of short contigs is an interesting problem. While EvoMIL could be applied to this setting in principle, we expect it to be less effective with the current configuration. The main challenge is that including viral contigs may result in a smaller

number of proteins useable in multiple instance learning, making the training unreliable. Furthermore, for short contigs, fewer proteins will also reduce the chance of including any of the key proteins that facilitate the interactions, making EvoMIL vulnerable to noise, but testing these hypotheses is beyond the scope of this work.

4. As described in the manuscript, it is noted that EvoMIL is currently designed to handle host prediction for a specific set of 15 prokaryotes and 5 eukaryotes and cannot perform well on other microbes. This means that if the dataset includes viruses that do not infect these specific microbes, EvoMIL may not be able to provide accurate predictions. To address this limitation, I would suggest that the authors consider introducing a class labeled as 'Other' to accommodate users who may wish to utilize EvoMIL with datasets containing viruses that infect organisms outside of the predefined set. This would allow for more flexibility and usability of EvoMIL in a broader range of scenarios.

We acknowledge that the number of hosts is low. We are not claiming to have trained a universal predictor. Instead, we demonstrated a new method that could train predictors. The hosts tested in our work include commonly studied species such as human, mouse, and E. coli, therefore benefiting a large community. Our training framework will allow researchers interested in other species to train their specific classifiers.

While adding an “other” could be done by retraining the classifier with the additional “other ” label, we are concerned with the effect of balancing the size of this label with others. We would have to bundle a large number of diverse viruses (mostly likely interacting with different hosts) into one category. This could significantly corrupt the signal for actual host labels. In principle, one could envision the classifier to pick up features that are different between the selected hosts and “other”, in reality, there are likely to be common features among viruses, and hosts in the “other” category might be evolutionarily related to the selected hosts. Finally, we selected the host based on the fact that those “other” hosts do not have enough information for EvoMIL. We, therefore, conclude that adding an “other” label would reduce the usability of the trained model.

5. The creation of the negative set:
To discuss more comprehensively, the authors provided 2 strategies to create the negative set: 1) The hosts of positive viruses and negatives viruses should not be in the same genus. 2) The natural hosts of positive viruses and negatives viruses are in the same taxonomic group (from genus to phylum). The author claims that the strategy 2 introduces challenging cases to evaluate the tool. It is partially correct. My concern is that two viruses respectively infecting rodent and human (both order Mammalia) can be

very different. If the similarity between them is very low, the case is then not that challenging.
In a typical bioinformatic scenario, creating the dataset by the sequence's similarity is preferred. A visualization of the similarity between the positive set and negative set could help mitigate the concern.

We have added the sequence similarities between the positive and negative sets in strategy 2. We further separated them in terms of taxonomy levels (genus, family, order, class, and phylum). The results are listed in the following figure. Most of the pairs have a similarity score larger than 0.6. We hope this mitigates the concern of easy cases. (see line 153)

We have added the following text to our paper:

"To quantify the hardness of the task, we computed the sequence similarity scores between all pairs of positive and negative sets on strategy 2 using MMSeq2 (Figure S2 and S3). Most of the scores are above 0.6. Looking at the scores across taxonomy levels, the phylum (purple) level tends to have lower similarities, while the family (orange) and order (green) levels tend to exhibit higher identity scores. These suggest a good degree of sequence similarity between positive and negative viruses, therefore a challenging classification task."

Figures for both prokaryotes and eukaryotes hosts are added to supplementary figures S2 and S3. Here we show the Figure S3 (eukaryotic hosts):

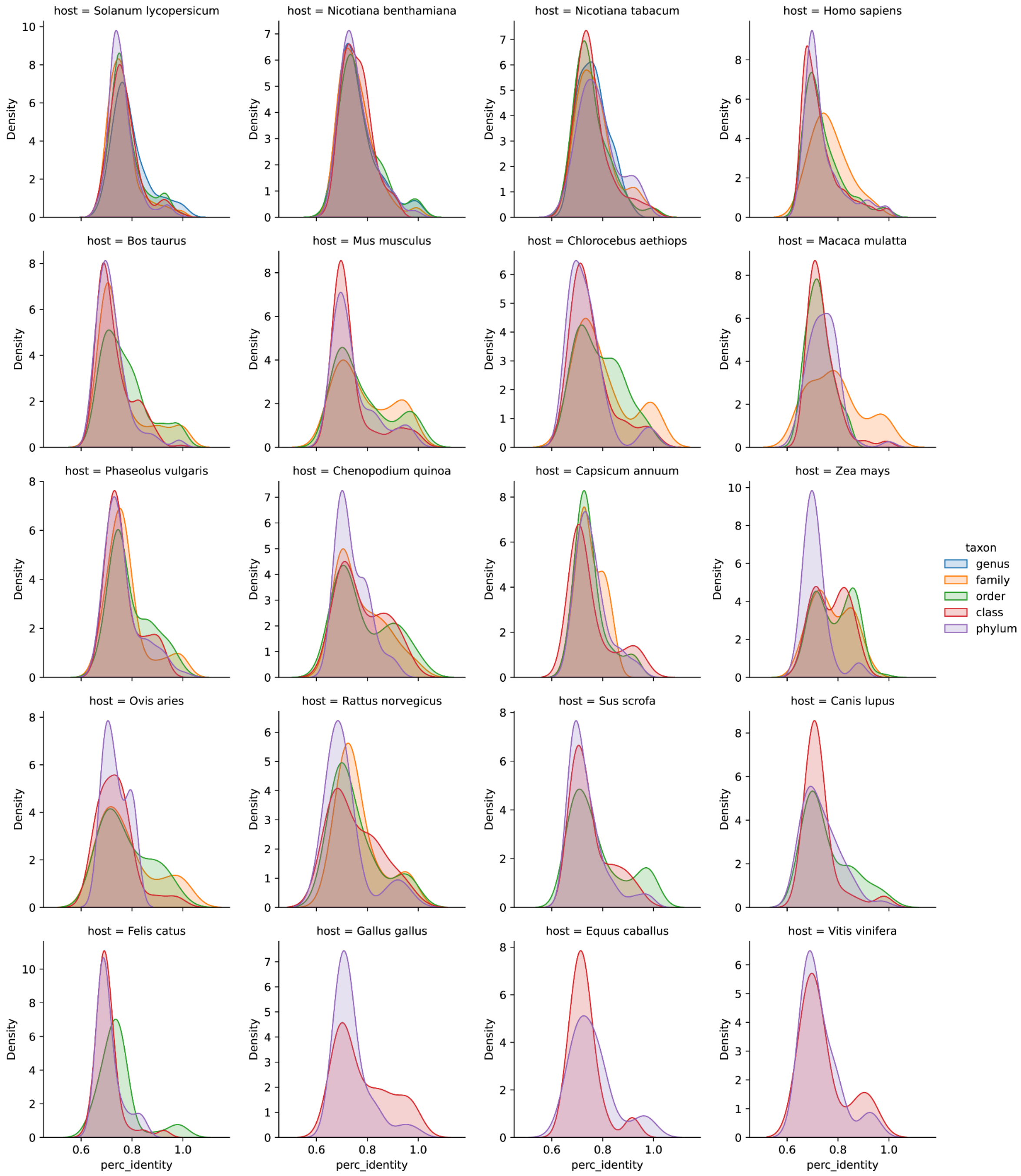

Fig S2. The genome sequence similarities between positive and negative viruses from different taxonomies on each eukaryotic host. This figure presents the distribution of genome similarities between positive and negative samples, where the negative viruses are chosen based on the same taxonomy (genus, family, order, class, phylum) as the positive viruses.

6. Number of the eukaryotic hosts.
In the first experiment (binary classification), the authors only created 5 eukaryotic datasets, because they require that there are at least 125 viruses in the positive set. The cutoff is a bit stringent. How will this method perform when the cutoff is 50 as the prokaryotic one? When the tool is designed on 36 eukaryotes, why not show them all?

The cutoff is set to ensure robust data for training MIL. A cutoff of 50 for eukaryotic would be too low, due to the fact that viruses for eukaryotic hosts have much fewer proteins (2-23) compared to those for prokaryotic hosts (45-212). Setting 50 would lead to not enough proteins across viruses to train a usable model. Empirically, we tested Gallus Gallus, the 6th eukaryotic dataset with the most viruses (109) among the 36 eukaryotes, the trained model has close random test AUC performances. Further down the list of eukaryotes, we observed highly variable AUC performance. We therefore excluded these datasets and models. As a comparison, the AUC performance for prokaryotic hosts is stable as reported in the paper.

We refer the reviewer to the following text in line 58 of the main text for a discussion about the cutoff selection: "The prokaryote datasets consist of nearly all dsDNA viruses which have 45 to 212 proteins coded in their genomes, while the eukaryotic datasets include many RNA viruses that contain fewer proteins, ranging from 2 to 23 protein sequences. The performance of MIL improves with higher numbers of instances in each bag, therefore we need to increase the threshold for the number of viruses in the eukaryotic training datasets to achieve similar performance with MIL. Accordingly, we set a threshold for minimum positive dataset size to 50 and 125 viruses for constructing prokaryotic and eukaryotic binary datasets, respectively. The aim of setting the threshold is to generate a sufficient number of training samples for prokaryotic and eukaryotic hosts, respectively."

Other detailed comments can be found below.
(1) The data distribution
Compared to the emergent novel viruses, the VHDB database is not complete. Although using it as a reference is acceptable, the authors should show the virus taxonomy distribution to the audience.

We added the virus taxonomy distribution to supplementary Figure S1. We have also added the following text in the discussion about this bias as a limitation of this study.

Line 418: "Additionally, we acknowledge that Virus-host DB is biased towards well-studied organisms such as humans, to mitigate this, combining different virus-host

association databases could enhance the diversity and comprehensiveness of the training samples.”

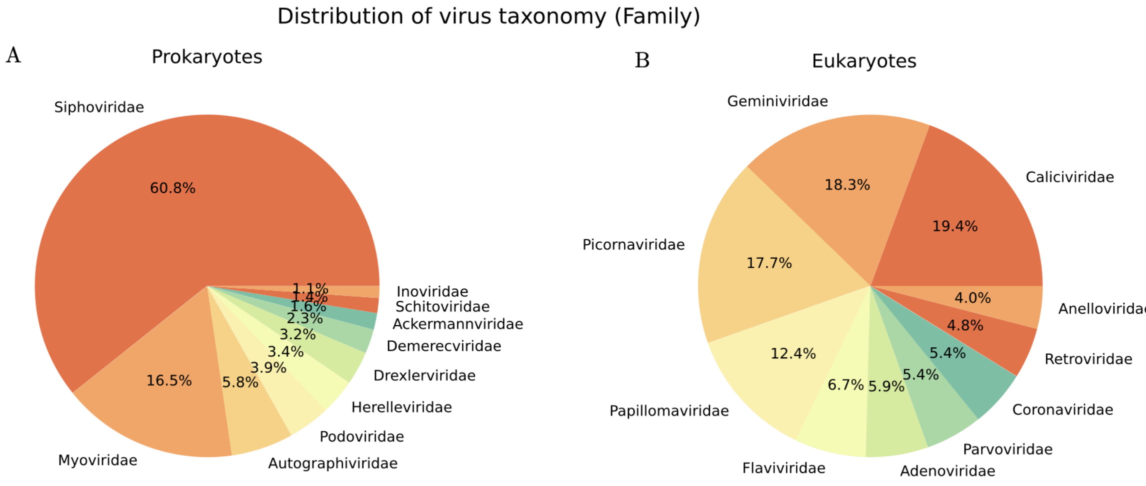

Fig S1. Virus taxonomy (family) distribution on prokaryotic (A) and eukaryotic (B) hosts. To illustrate the virus taxonomy distribution, we plot pie charts to show the distribution of virus families in prokaryotic and eukaryotic hosts. Viruses associated with prokaryotes are dominated by Siphoviridae family, which constitutes approximately 60% (A), whereas the Geminiviridae, Picornaviridae and Papillomaviridae families are the top three ranked families, each accounting for roughly 18% (B).

(2) Model structure

Although the attention deep MIL module is from an existing work, the authors still need to elucidate the detail of the module. In Fig 1, the structures of neural network and the final classifier are not mentioned.

We have added details about the neural network and the final classifier to Fig. 1c (see below), and a more detailed text description of attention-based MIL in the Materials and Methods section.

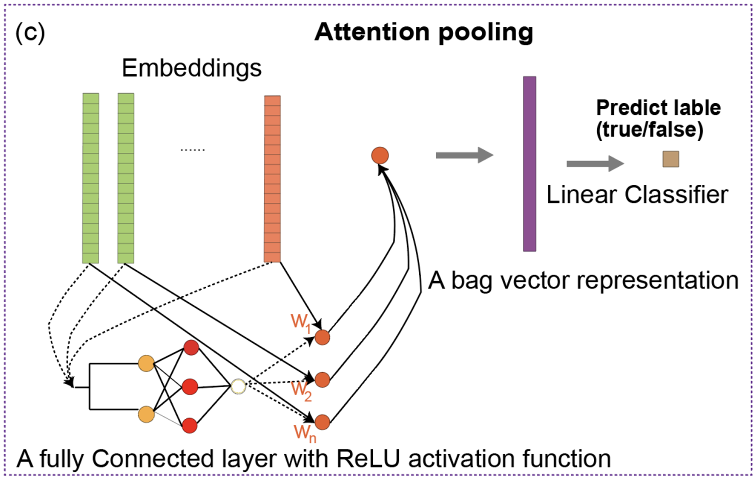

We have added the following revised text to our paper**:**

line 550: ”φ is a neural network that transforms protein embedding to a lower dimensional space. φ is implemented as a fully connected layer with ReLU activation functions. It has an input size matching embedding dimension from ESM-1b and k-mers, and an output size universally set to 800.”

The learning architecture of AA_2, PC_4, and DNA_5 is not stated.

The same MIL learning architecture used for all feature sets including AA_2, PC_4, and DNA_5, was applied the same way as for the ESM-1b embeddings. We have added details about k-mers AA_2, PC_4, and DNA_5 to the section “Materials and Methods” (see line 520).

(3) Problem formulation
In the second experiment (multi-class task), is the data multi-label? Or with single label? This is not stated. The definition of “multi-class classification” and “multi-label classification” is different. While the former only outputs one label, the latter might output multiple labels for one query. And a number of viruses can infect multiple hosts. Without stating this, the problem formulation is not finished.

We have further clarified our model as a multi-class classification in section 2.3. See line 160 for the updated section title, and the text from the paper below:

“2.3 Embedding features outperform protein and DNA k-mer features on multi-class classification tasks

To demonstrate that the ESM embeddings are encoding highly predictive information than conventional features, we generated feature sets from the k-mer composition of

the nucleic acid, amino-acid and physio-chemical sequences, and implemented ESM-1b and k-mer features on multi-class classification model."

(4) Fig 3 and Table 5
The caption of Fig.3 is not clear. Which experiment does it belong to? It seems that Fig 3 (a, c) and Table 1 are collectively for 22 prokaryotes and 36 eukaryotes. And Fig 3 (b, d) is for small dataset. The organization is interleaved. It is hard to follow the logic.

We are sorry for the confusion. We have revised Fig. 3 and clarified the text in the caption.

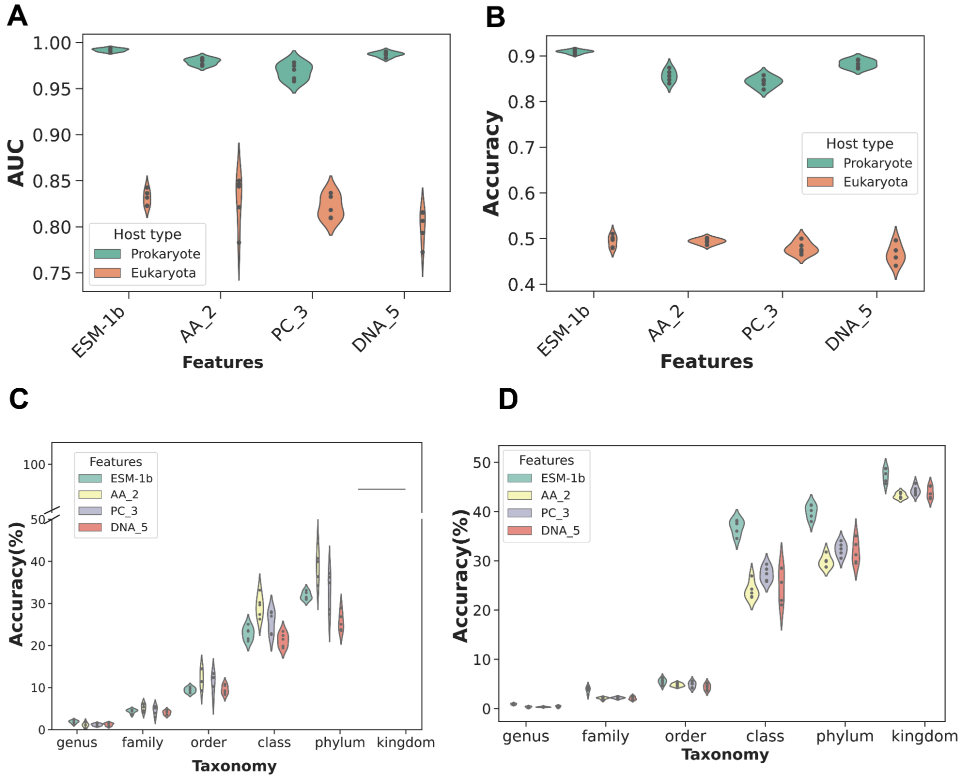

Fig 3. A and B represent the AUC and accuracy, respectively, for prokaryotic and eukaryotic hosts using four feature sets (ESM-1b, AA 2, PC 3 and DNA 5), AUC and accuracy are equivalent with those presented in Table 1. C and D indicate the results obtained by testing the trained models on prokaryotic and eukaryotic hosts associated with 5 to 30 viruses, using the four different feature sets described above.

(5) Number of the prokaryotic hosts.
The author only included 22 prokaryotes, which is rather limited.

The rationale for selecting 22 prokaryotes is the same as eukaryotic hosts. These are the hosts with enough viruses and protein for training a reliable classifier.

(6) The accuracy of multi-classification.
The tool is finally complied as a multi-class classifier among 36 eukaryotic hosts. And according to table 1, the accuracy on test set is around 49.4%. This is not a satisfactory result. I suppose the alignment-based method like BLASTn can achieve this as well.

The multi-class classification model on 36 eukaryote hosts is 49.4%, which is significantly higher than the accuracy of a random classifier with 36 classes of 2.8% (1/36 x100).

We attempted to implement BLASTn-based classifier, however, BLASTn cannot score viruses with eukaryotic hosts.

(7) Fig 5 and Fig S2

In Fig 5, is true label at the left axis? This is not stated.
While Fig 5 shows the error cases in each host group and different rank by heatmap, Fig S2 uses the confusion matrix directly. They are of a bit redundancy. I recommend the authors use Fig S2 in the main part instead of Fig 5. Because Fig 5 does not reflect the error trend.
In Fig S2, most of the area is white, because a strong signal is at Homo-Homo (212). To better show the result, I strongly recommend the author conduct row normalization by the actual classes.

Yes, it is the true labels at the left axis in Fig. 5, we added “actual classes” on the left axis.

We followed this suggestion and updated Fig 5. We show Fig. 5A below, and the original Fig. 5 was moved to Fig S4.

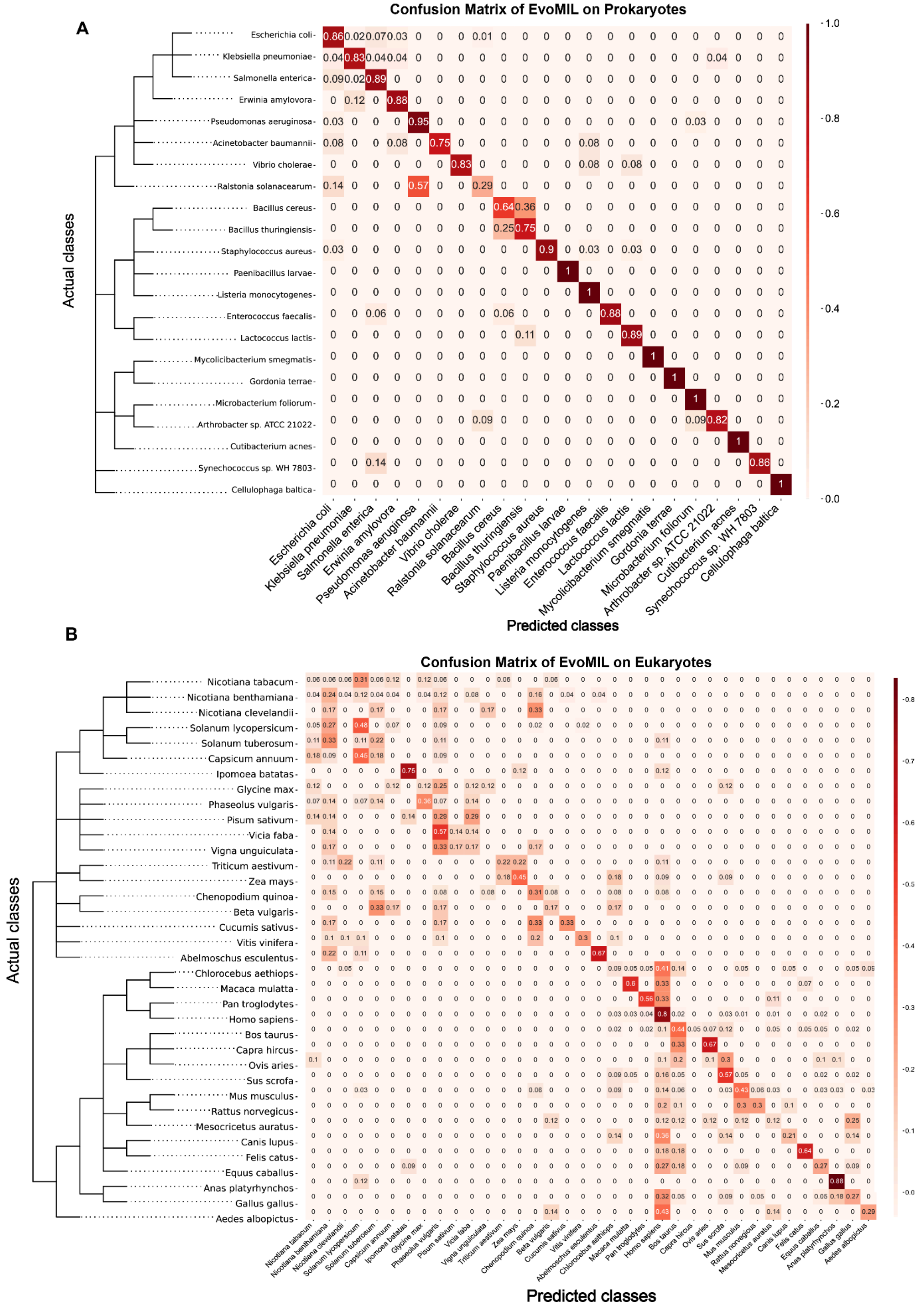

Fig 5. The Confusion matrix plot of prokaryotic hosts (A) and eukaryotic hosts (B) based on EvoMIL. The confusion matrix plots A and B represent the performance of

the EvoMIL model on 22 prokaryotic hosts and 36 eukaryotic hosts, respectively. It is constructed by evaluating the model's predictions on a test set comprising 20% of the dataset, while the EvoMIL model was trained on the remaining 80% of the data. This plot provides insights into the model's accuracy in predicting the host species for the tested viruses.

(8) Balanced binary dataset
In the part of the balanced binary dataset, the author defined 4 vectors, V, Vpos, Vneg, and Vpro. V is of P length, and Vpro is of T length. T is not defined, so that Vpro is confusing as well.
In 450, there is a typo “Using this method”.

We defined T, here Vpro meaning all viruses associated with prokaryotes.

We have added the following revised text to our paper:

line 462: “As for a given host H in the prokaryotic datasets, all viruses are represented by Vpro={V1, V2,...VT}, where T is the total number of viruses associated with prokaryotic hosts, we collect negative viruses Vneg = Vpro-Vpos, which are not related with the given host; and host species taxonomy of V_neg are different from host H.”

When we look at the virus world from an individual host, like human, most of the viruses in the world do not infect human. So, I think it is better to include all the negative data, instead of just using a small part of it.

The reasons why we did not include a larger negative set are the following:

1. Using all available negative data could result in a highly imbalanced dataset, this imbalance can lead to biased model training and cause the model to mainly learn from negative samples. Therefore, subsampling from virus-host pairs with unknown associations becomes the only scalable option in the absence of a curated database for negative virus-host associations.
2. Previous host prediction tasks for binary classification have also used balanced datasets for training and evaluation[1]. This method has proven effective in obtaining interpretable results.

[1] Young F, Rogers S, Robertson DL. Predicting host taxonomic information from viral genomes: A comparison of feature representations. PLoS Computational Biology. 2020 May 26;16(5):e1007894.

(9) The UMAP section

The authors want to know “whether the embeddings of these important proteins identified by attention-based MIL contain any underlying clustering structures.” But the clustering structures can only show the successful application of ESM-1b embedding without any further benefit to the host part. As long as the proteins number is large enough, there definitely will be clusters in UMAP. However, ESM-1b is not contributed by this work. So, I don’t understand why they put this here.

We agree with the reviewer about the added value of UMAP. Our goal is to contextualise the important proteins picked up by EvoMIL. We have now provided the number of GO term hits in the highly weighted proteins (Figure 7). The clustering structure is indeed not a novel result of our method, however, we still think this observation is useful in explaining the predictive power of EvoMIL. That is ESM-1b encodes functional similarities of different proteins, and one could use ESM embedding as a common feature set to train an effective classifier. We now provide supplementary material hierarchical clustering results (Fig. S5) demonstrating the clustering of protein hits with the same GO terms. We have removed the UMAP figures.

In 324th row, please try to explain the difference.

We apologise for the confusion. We have removed the corresponding discussion.

Reviewer #2: >>> Overall, ESM-1b demonstrated superior performance compared to the k-mer features, through 5-fold cross-validation on both prokaryotic and eukaryotic hosts. Furthermore, we split multi-class datasets into 80% training and 20% test sets, then train and test our model without cross-validation, and compare the accuracy for each host to evaluate the prediction performance of ESM-1b and k-mer features on multi-class classification.
I don’t understand why this would be desirable if you already have the results of a 5-fold cross validation. I’d remove this part and make Figure 4 be based on the results from the cross validation (i.e., have the log2(esm-kmer) values be computed for each fold separately and then provide standard deviation markers on the barplots. Also, for this figure please provide guidelines or better spacing between each set of bars to make it easier to read which set of bars correspond to each species

We agree with this suggestion. Figure 4 has been updated, please see below and Line 227.

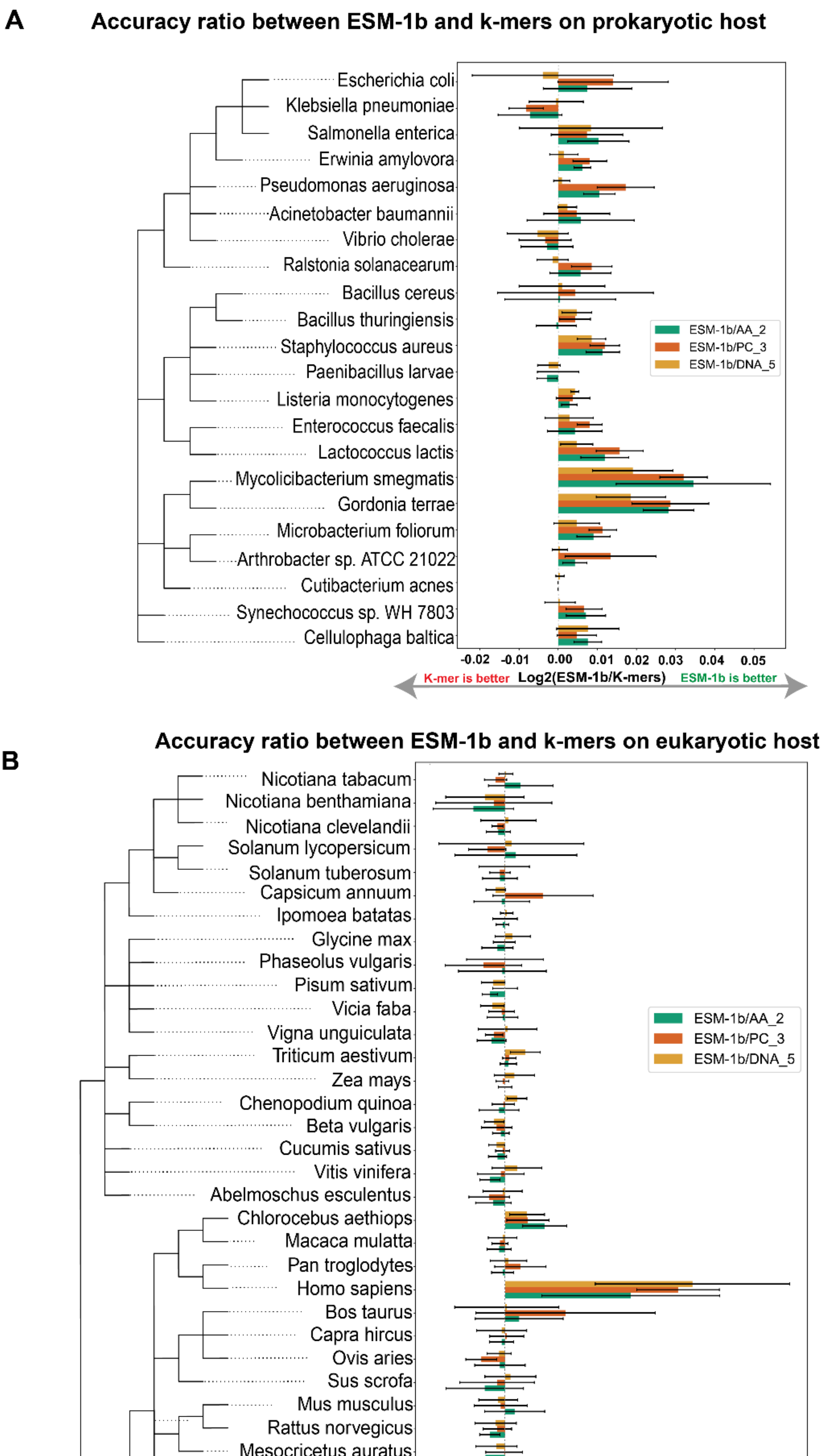
A
Accuracy ratio between ESM-1b and k-mers on prokaryotic host
Escherichia coli
Klebsiella pneumoniae
Salmonella enterica
Erwinia amylovora
Pseudomonas aeruginosa
Acinetobacter baumannii
Vibrio cholerae
Ralstonia solanacearum
Bacillus cereus
Bacillus thuringiensis
Staphylococcus aureus
Paenibacillus larvae
Listeria monocytogenes
Enterococcus faecalis
Lactococcus lactis
Mycolicibacterium smegmatis
Gordonia terrae
Microbacterium foliorum
Arthrobacter sp. ATCC 21022
Cutibacterium acnes
Synechococcus sp. WH 7803
Cellulophaga baltica
ESM-1b/AA_2
ESM-1b/PC_3
ESM-1b/DNA_5
-0.02 -0.01 0.00 0.01 0.02 0.03 0.04 0.05
K-mer is better
Log2(ESM-1b/K-mers)
ESM-1b is better
B
Accuracy ratio between ESM-1b and k-mers on eukaryotic host
Nicotiana tabacum
Nicotiana benthamiana
Nicotiana clevelandii
Solanum lycopersicum
Solanum tuberosum
Capsicum annuum
Ipomoea batatas
Glycine max
Phaseolus vulgaris
Pisum sativum
Vicia faba
Vigna unguiculata
Triticum aestivum
Zea mays
Chenopodium quinoa
Beta vulgaris
Cucumis sativus
Vitis vinifera
Abelmoschus esculentus
Chlorocebus aethiops
Macaca mulatta
Pan troglodytes
Homo sapiens
Bos taurus
Capra hircus
Ovis aries
Sus scrofa
Mus musculus
Rattus norvegicus
Mesocricetus auratus
ESM-1b/AA_2
ESM-1b/PC_3
ESM-1b/DNA_5

Fig 4. The taxonomy tree, aligning with Log2 of ratio accuracy between ESM-1b and k-mers. The figure shows the taxonomy tree of 22 prokaryotic (A) and 36 eukaryotic (B) hosts. Each host is aligned with a bar plot showing the accuracy ratio and standard deviation of 5-fold cross-validation between ESM-1b and AA 2, PC 3, and DNA 5, respectively.

>>> "Similarly, ESM-1b outperforms the other feature sets in 16 out of 36 eukaryotes." Does this mean that your model failed to outperform a very simple baseline in most of the eukaryote datasets?

We have removed this confusing statement. The original statement was about Figure 4. The updated discussion can be found below:

line 227: "These findings indicate that ESM-1b outperforms k-mer feature sets in multi-classification tasks for prokaryotic hosts. For eukaryotic hosts, ESM-1b's performance is comparable to k-mer features in all eukaryotes except *H. sapiens.* Notably, ESM-1b demonstrated significant improvement over k-mer on H. sapiens, which has the largest number of training samples, indicating that EvoMIL performs better with a larger number of virus training samples for eukaryotic hosts.*"*

>>> Please make the methodological details of how the features for AA 2, PC 3 and DNA 5 were created for reproducibility.

We have added the details about how the AA-2, PC-3 and DNA-5 features were generated to the Methods section (see line 519).

We have added the following text to our paper:

line 519: "K-mer composition vectors were generated for each of the CDS regions of a virus and used as an alignment-free representation of a sequence. A kmer is a sub-sequence of the length of k and is generated for each position in a sequence. These features are obtained by calculating the frequency of each of the possible k-mers in a sequence. To minimise the effect of sequence length, the resulting vector is normalized so that its sum is equal to 1. Here, we extract k-mer features from sequences corresponding to DNA, amino acids(AA) and their physio-chemical properties (PC) [1]. DNA and amino acid sequences of the CDS regions were downloaded from the NCBI database. and used directly to extract AA and DNA k-mers. To extract PC k-mers from protein sequences we first re-label each amino acid as one of seven groups based on its physio-chemical properties: ({AGV}, {C}, {FILP}, {MSTY}, {HNQW}, {DE}, and {KR}), we then extract k-mers using the seven group labels as the alphabet. To generate kmer composition vectors of reasonable length for MIL we use

kmer lengths of 5, 2 and 3 respectively for the DNA, AA and PC sequences. This results in DNA_5, AA_2, PC_3 feature sets of dimensions of 1024, 400, and 343 respectively."

[1] Young F, Rogers S, Robertson DL. Predicting host taxonomic information from viral genomes: A comparison of feature representations. PLoS Computational Biology. 2020 May 26;16(5):e1007894.

>>> It would be nice if Figure 5 had a taxonomy tree similarly to Figure 4

We take this suggestion, the taxonomy tree is added and moved to supplementary Figure S6 after considering the comments from reviewer#1.

>>> Please make Figure 6 have a white background to save ink if someone ever prints this.

We have removed the old Figure 6 (UMAP plots).

>>> "Again, the UMAP plots demonstrate that different protein functions are clustered into separate locations, while GO terms with similar functions tend to form sub-clusters." I disagree with this statement, it seems that there are many proteins with the same GO term that are separated in different clusters (e.g. "Virion Assembly" in subfigures A, B and "Viral Assembly" subfigures C,D). If you want to make this claim please run a clustering algorithm (k-means will suffice, but a hierarchical clustering algorithm would give you better working space to make claims) and see to which degree certain functions cluster or do not cluster together.

We revised the discussion about clusterings. Following the reviewer's suggestion, we used hierarchical clustering to cluster top-ranked proteins based on their ESM-1b embeddings. We found apparent clusters of proteins with common GO terms, which explains the predictive signal captured in ESM-1b. We also observed that not all proteins with the same GO terms are assigned to single clusters. This suggests that ESM-1b embedding may contain information beyond known function annotations that are helpful for predictions. We feel these insights, while not being methodology innovations, are useful for readers to better understand how EvoMIL works.

In addition, we agree with the reviewer that the UMAP doesn't add much to the paper. We have now removed the UMAP figure.

Please see the supplementary Figure S5 and line 342 for descriptions, and the revised text is copied below:

Hierarchical Clustering Dendrogram of viral proteins associated with Escherichia coli on Binary model (A) and Multi-class model (B)

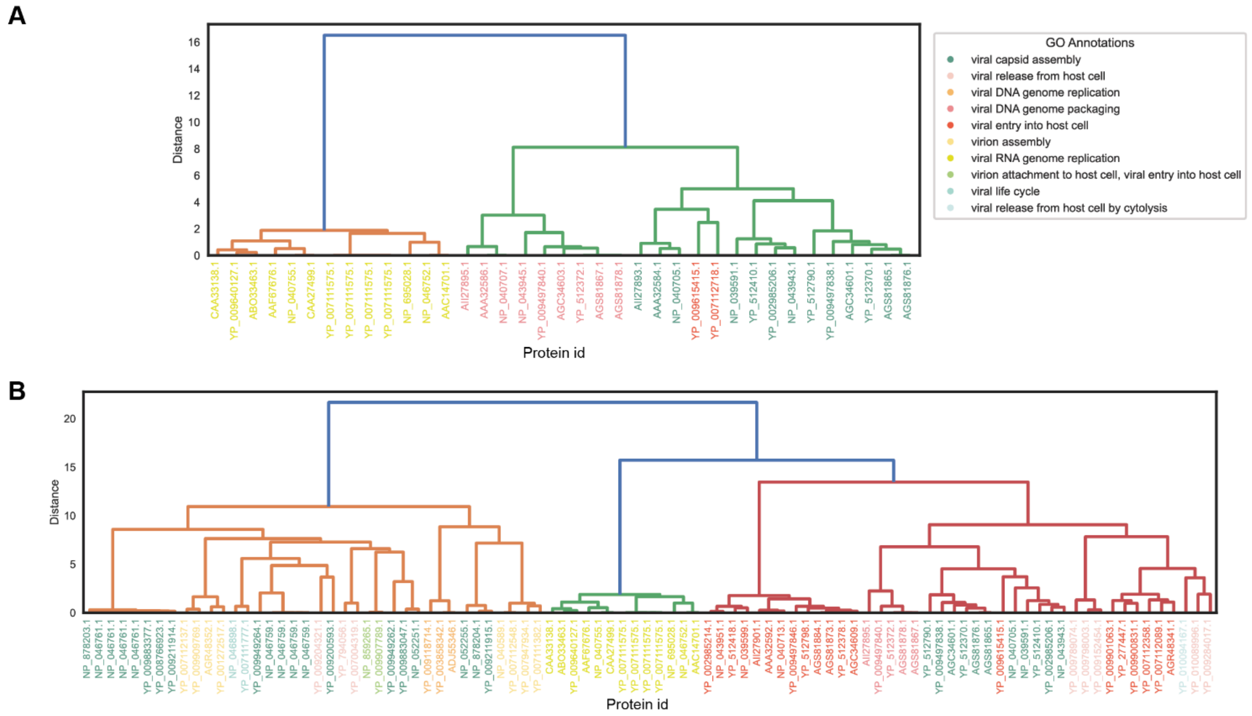

Hierarchical Clustering Dendrogram of viral proteins associated with Homo sapiens on Binary model (C) and Multi-class model (D)

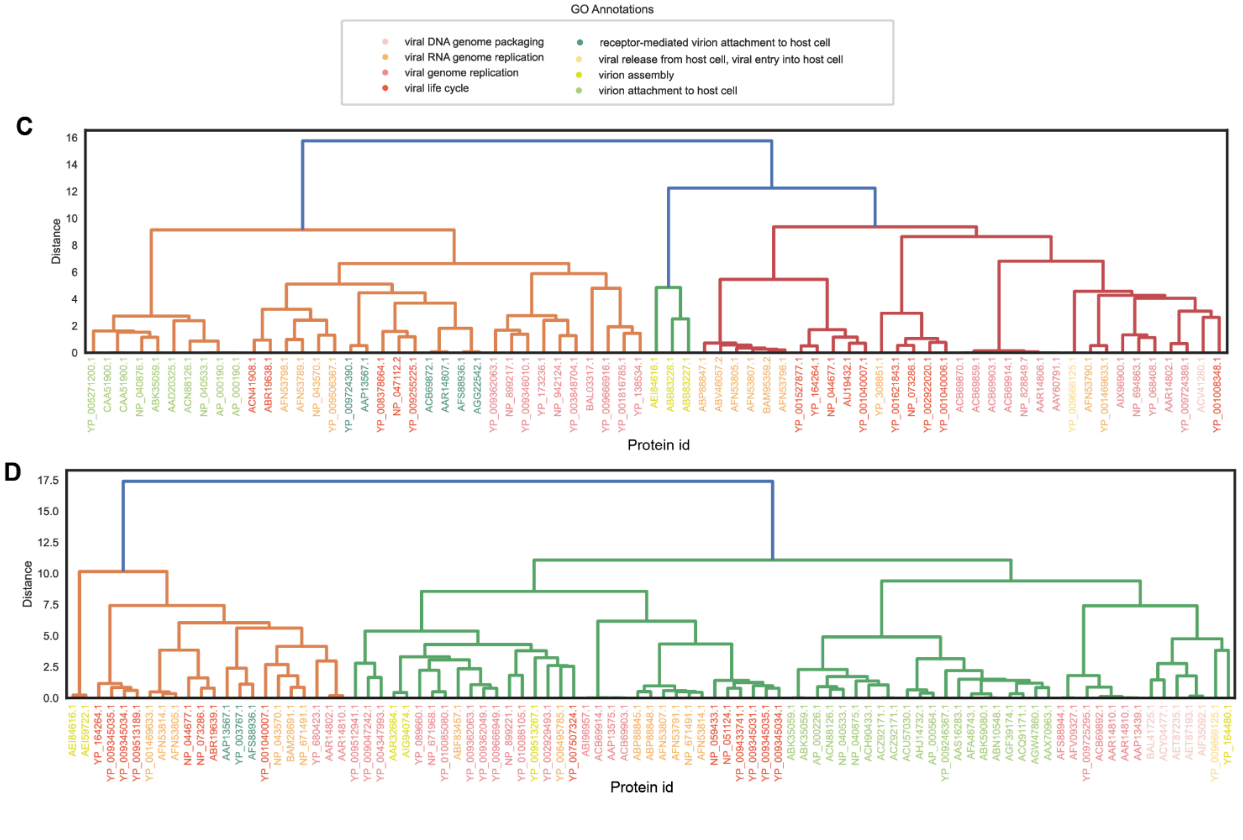

Fig S5. The dendrogram of protein embeddings of viruses associated with *E. coli* (top) and *H. sapiens* (bottom). Figures show the hierarchical clustering dendrograms of the top 5 ranked protein embeddings for each virus associated with *E. coli* (A, B) and *H. sapiens* (C, D). Here, the protein weights in A and C are obtained by binary models, whereas in B and D, the weights are obtained by multi-class classification models. Protein GO terms regarding the viral life cycle of viruses are highlighted with different colours, allowing us to understand the predictive signals of proteins captured by the pre-trained transformer model.

We have added the following text to our paper:

Line 342: "Furthermore, we used hierarchical clustering to test if different proteins with the same functional annotation have similarities in the embedding space. In Figure S5 we clustered the ESM-1b embeddings of the abovementioned top-ranked proteins with viral life cycle GO annotations. We found apparent clusters across the viral proteins for *E. coli* (Figure S5 A, B) and *H. sapiens* (Figure S5 C, D). These results suggest ESM-1b embeddings encode functional signals, and MIL learns some consistent features associated with host specificity. While having clusters of proteins with the same GO annotation, we also observe that not all proteins with the same GO annotation are clustered into a single cluster. This suggests that there is richer information contained in the embedding space beyond existing function annotations. Whether this information is biologically meaningful warrants a future study."

>>> "For example, the indices of GO annotations of the top 5 ranked proteins (Fig 7 A) are the same as the indices of all proteins (Fig 7 B), meaning that we can obtain GO annotations although only selecting top 5 ranked proteins."
This sentence is a bit confusing, although one can understand it after referring to the image. It would be good if you could make a bit more time to make this sentence clearer.

We have rewritten this sentence and have added the following revised text to our paper:

Line 301: "We mark the GO annotation for proteins and select three GO annotations as examples. For example, the top 5 ranked proteins (Fig 8 A) and all ranked viral proteins (Fig 8 B) associated with E. coli shared the same GO annotation (viral capsid assembly) and have identical rank index within their respective ranked protein sets, meaning that selecting the top 5 ranked proteins of each virus still allows us to identify key GO annotations."

Reviewer #3: In this paper the authors present an efficient model able to carry out the virus-host association starting from the proteins set of the virus.
This is a novel method and all the presented data and analysis support the claims.
Some more details are missing about some theoretical concepts, methods or technical issues.
The code and the trained models are available on a GitHub link on the paper and the

original data are collected from the Virus-Host database. Nevertheless, the descriptions of the algorithm, the dataset and the used architectures are not satisfactory in the paper for the sake of reproducibility.
The paper has been written with a good and clear english but some concepts are hardly accessible for non-specialists.

In conclusion I suggest to extend some part of the paper in order to make it more accessible to non-specialists and also make the experiments easily reproducible.
You can find below some questions that could be useful for this task:
- Can you explain in a more clear and detailed way the so called "strategy 1" and "strategy 2" ?

Strategy 1 and **Strategy 2** are two ways to choose negative samples. we add more details about these two strategies (see line 74).

We have added the following revised text to our paper:

Line 74: We used two different strategies to sample the negative viruses from those with no known association with each host identified above to create balanced binary datasets. Given that the actual association is unknown, this is susceptible to false negative labels.

"Strategy 1" was used to establish the concept of EvoMIL. We sampled the negative viruses from all viruses that are in different genera than viruses in the positive dataset, with the aim of minimising the false negative viruses in the dataset.

"Strategy 2" aimed to make the task progressively more challenging using the fact that as a result of coevolution and co-speciation similar viruses tend to infect similar hosts. Here we selected the negative viruses from those that infect hosts in the same taxonomic rank as the positive host, from phylum to genus, thereby meaning the classifier had to distinguish between more and more similar viruses.

- What's the aim of your binary and multi-class classification models?

The binary classification models aim to demonstrate the predictive power of EvoMIL while analysing the influence of negative sample selection methods. How to select negative samples is a common challenge in the virus-host prediction task.

The aim of the multi-class classification models is a more realistic setting of the virus-host prediction task. Currently, more predictors could pinpoint the importance of single proteins in virus-host prediction without substantial post-hot analysis. Our model addresses this important gap, in a realistic application setting.

- Can you explain and describe a little more the K-mer features?

We added the details about K-mer features to the Materials and Methods section (see Line 519).

We have added the following text to our paper:

line 519: “K-mer composition vectors were generated for each of the CDS regions of a virus and used as an alignment-free representation of a sequence. A kmer is a sub-sequence of the length of k and is generated for each position in a sequence. These features are obtained by calculating the frequency of each of the possible k-mers in a sequence. To minimise the effect of sequence length, the resulting vector is normalized so that its sum is equal to 1. Here, we extract k-mer features from sequences corresponding to DNA, amino acids(AA) and their physio-chemical properties (PC) [1]. DNA and amino acid sequences of the CDS regions were downloaded from the NCBI database. and used directly to extract AA and DNA k-mers. To extract PC k-mers from protein sequences we first re-label each amino acid as one of seven groups based on its physio-chemical properties: ({AGV}, {C}, {FILP}, {MSTY}, {HNQW}, {DE}, and {KR}), we then extract k-mers using the seven group labels as the alphabet. To generate kmer composition vectors of reasonable length for MIL we use kmer lengths of 5, 2 and 3 respectively for the DNA, AA and PC sequences. This results in DNA_5, AA_2, PC_3 feature sets of dimensions of 1024, 400, and 343 respectively.”

[1] Young F, Rogers S, Robertson DL. Predicting host taxonomic information from viral genomes: A comparison of feature representations. PLoS Computational Biology. 2020 May 26;16(5):e1007894.

- Can you add a few line about UMAP, Gene Ontology, GO annotation and InterProScan?

We removed the UMAP plots, so we added Gene Ontology, GO annotation, and InterProScan to the Gene Ontology section (see line 557).

We have added the following text to our paper:

Line 557: “Gene Ontology (GO) is used to describe gene function from different organisms, which includes three GO domains: biological process, cellular component and molecular function. GO annotations are links between gene products and GO terms, and the structure of GO can be represented by a graph, each node is a GO term and nodes are connected by edges. InterProScan is a tool to obtain GO terms by classifying query proteins to protein families within InterPro's database. To analyze the

protein functions of important virus proteins identified by EvoMIL, we used InterProScan to obtain GO annotations of viruses."

- Can you describe the used deep learning architectures and the input data dimensions in order to make possible the reproducibility of your experiments?

For deep learning architectures see the Methods section (line 550). For the details about input data dimensions see lines 492 and 533.

We have added the following revised text to our paper:

line 550: "φ is a neural network that transforms protein embedding to a lower dimensional space. φ is implemented as a fully connected layer with ReLU activation functions. It has an input size matching embedding dimension from ESM-1b and k-mers, and an output size universally set to 800."

line 492: "EvoMIL is based on a fully connected layer with an input size of 1280 (ESM-1b embeddings size) and an output size 800, and then using a linear classifier and the output size is the number of prediction classes."

line 533: "These AA_2, PC_3, DNA_5 features were used as inputs for the Attention-based MIL learning tasks, and the feature dimensions are 400, 343 and 1024, respectively."
