# Supplementary material for: Prediction of virus-host associations using protein language models and multiple instance learning
Source: PLoS Comput Biol. 2024 Nov 19;20(11):e1012597. doi: 10.1371/journal.pcbi.1012597 (PMC11614202; doi:10.1371/journal.pcbi.1012597)
Supplement: Attachment — Submitted filename: PLOS comp bio 2nd revision point to point reply.pdf [file pcbi.1012597.s016.pdf]

Point-to-point reply to reviewers

We thank the reviewers for a second round of insightful review comments. Please find below for the point-to-point reply.

Reviewer #1: The authors have addressed some of my comments, which I believe has improved the manuscript. However, I still think the authors are able to add BLAST (or BLASTp) as a benchmark method. This is important to examine the difficulty of the problem. Although the authors mentioned there is no published method for this, it is quite feasible. First, if the contigs contain multiple proteins, a simple **majority vote** can be applied. Authors can choose the variants of the majority vote strategy for this. Second, the authors can use BLASTn and do not need to rely on proteins. Nevertheless, this experiment does not need very complicated design. Instead, it will provide useful insights.

Re: Thank you for raising this point again. We have now implemented a BLASTp baseline as suggested. The benchmarking result now is included in Figure 6. Please check the updated version below. Please note that we also have a BLASTn-based baseline result in Figure 6.

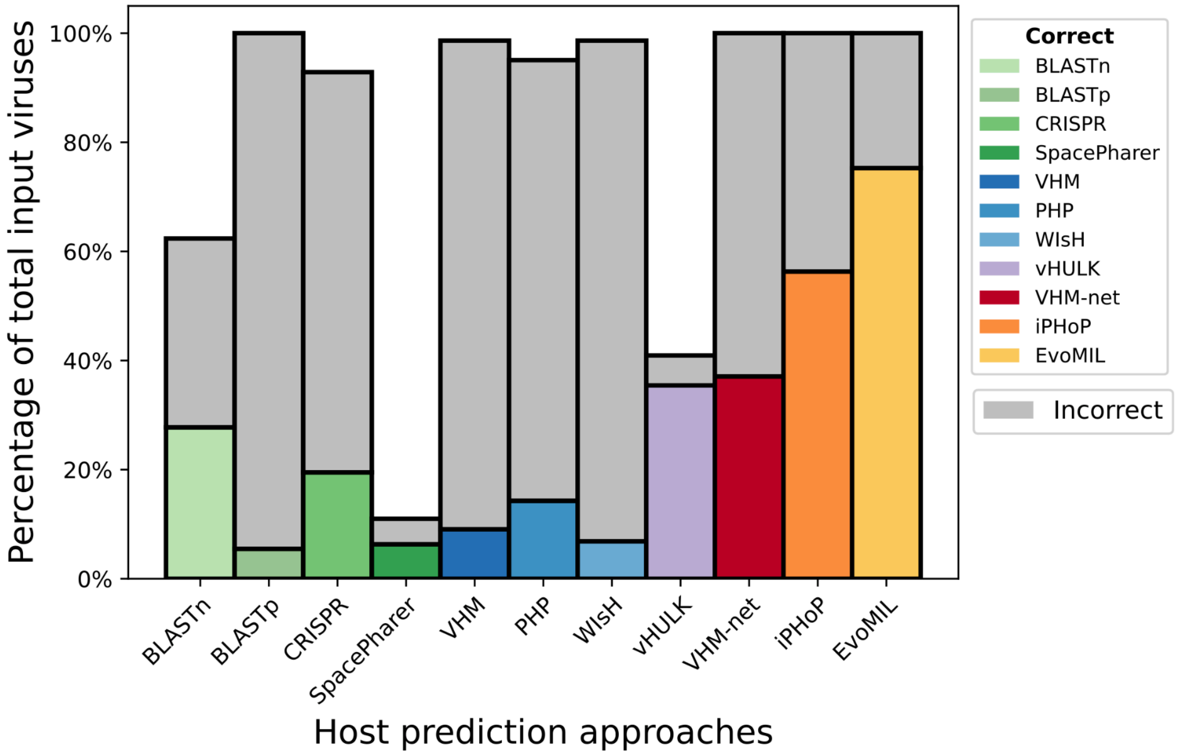

We added the following text to the Methods (see line 579 **):**

“BLASTp (v2.12.0+) was used to calculate the percentage of protein identity between the test virus and the prokaryotic host protein sequences from our training set, retrieved from NCBI. A maximum of 200 protein sequences per host were downloaded, and the host with the top-ranking match was selected as the predicted host.”

Reviewer #2: The authors seem to have addressed most of my points about the paper. Here are a few more suggestions and a typo:

there is no explicit definition of dsDNA

Thank you for your suggestion. We updated to “dsDNA (double-stranded DNA)”. See line 60.

predictiton -> prediction

Thanks, we addressed it.

Figure 4 is better now, it would also be good to have a **non-ratio** version in the supplementary.

Re: Thank you for your suggestion, we added the non-ratio version as Figure S4.

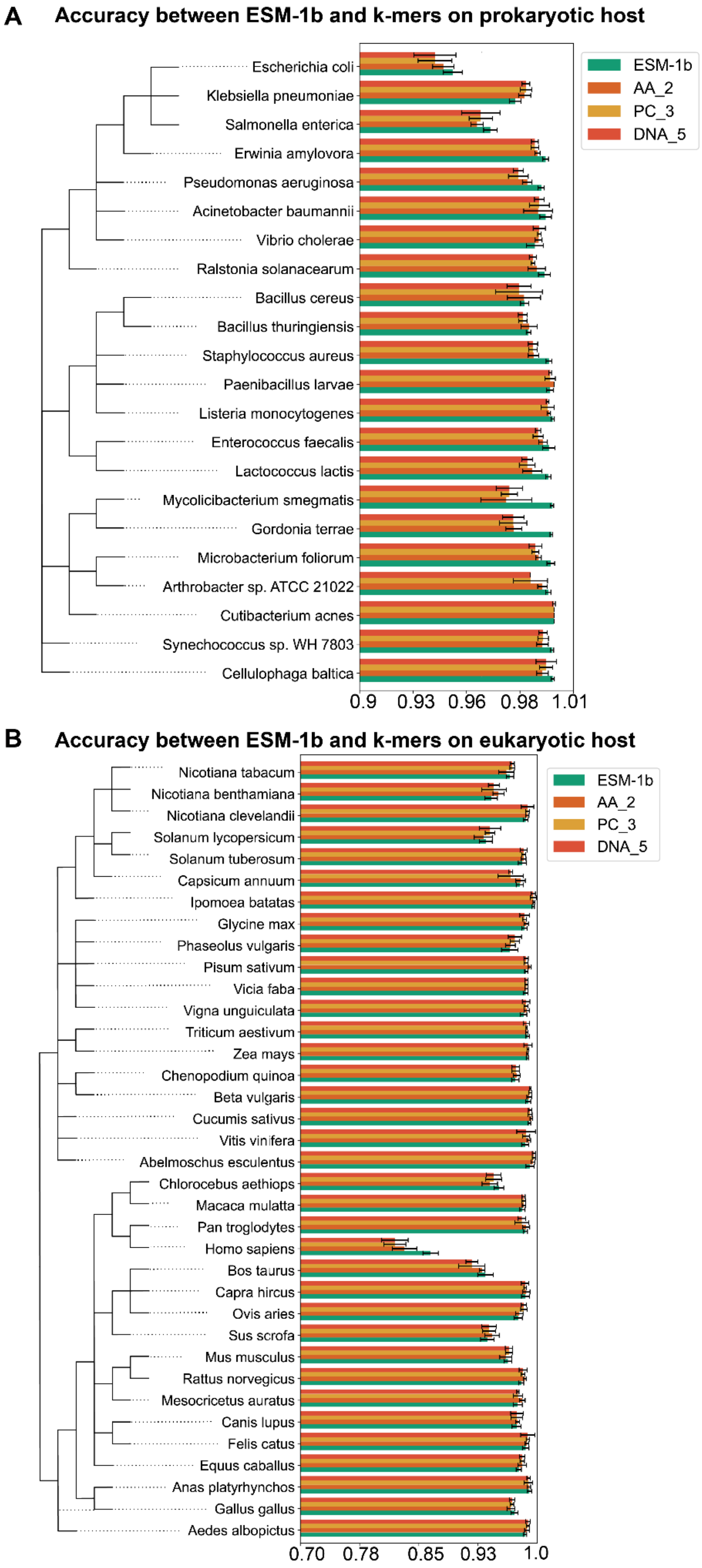

A
Accuracy between ESM-1b and k-mers on prokaryotic host
Escherichia coli
Klebsiella pneumoniae
Salmonella enterica
Erwinia amylovora
Pseudomonas aeruginosa
Acinetobacter baumannii
Vibrio cholerae
Ralstonia solanacearum
Bacillus cereus
Bacillus thuringiensis
Staphylococcus aureus
Paenibacillus larvae
Listeria monocytogenes
Enterococcus faecalis
Lactococcus lactis
Mycolicibacterium smegmatis
Gordonia terrae
Microbacterium foliorum
Arthrobacter sp. ATCC 21022
Cutibacterium acnes
Synechococcus sp. WH 7803
Cellulophaga baltica
0.9
0.93
0.96
0.98
1.01
ESM-1b
AA_2
PC_3
DNA_5
B
Accuracy between ESM-1b and k-mers on eukaryotic host
Nicotiana tabacum
Nicotiana benthamiana
Nicotiana clevelandii
Solanum lycopersicum
Solanum tuberosum
Capsicum annuum
Ipomoea batatas
Glycine max
Phaseolus vulgaris
Pisum sativum
Vicia faba
Vigna unguiculata
Triticum aestivum
Zea mays
Chenopodium quinoa
Beta vulgaris
Cucumis sativus
Vitis vinifera
Abelmoschus esculentus
Chlorocebus aethiops
Macaca mulatta
Pan troglodytes
Homo sapiens
Bos taurus
Capra hircus
Ovis aries
Sus scrofa
Mus musculus
Rattus norvegicus
Mesocricetus auratus
Canis lupus
Felis catus
Equus caballus
Anas platyrhynchos
Gallus gallus
Aedes albopictus
0.70
0.78
0.85
0.93
1.0
ESM-1b
AA_2
PC_3
DNA_5

Figure S4. The taxonomy tree, aligning with accuracy values between ESM-1b and k-mers. The figure shows the taxonomy tree of 22 prokaryotic hosts (A) and 36 eukaryotic (B) hosts. Each host is aligned with a bar plot showing the accuracy and standard deviation of 5-fold cross-validation between ESM-1b and AA 2, PC 3, and 675 DNA 5, respectively.

"Here, we extract k-mer features from sequences corresponding to DNA, amino acids(AA) and their physio-chemical properties (PC) [13]. (...) To extract PC k-mers from protein sequences we first re-label each amino acid as one of seven groups based on its physio-chemical properties: ({AGV}, {C}, {FILP}, {MSTY}, {HNQW}, {DE}, and {KR}), [30]" Although you cite [13] above as the citation for all the three characteristics, you only cite 30 at the end of the second sentence highlighted, it'd be good to have a more explicit citation mentioning that the physiochemical property grouping is from [30]. Not necessary, but would aid clarity.
[13] doi:10.1371/journal.pcbi.1007894
[30] doi:10.1073/pnas.0607879104

Re: Thank you for your suggestion, we revised this sentence (see line 529):

"Here, we extract k-mer features from sequences corresponding to DNA, amino acids(AA) and their physio-chemical properties (PC) following the paper [13]. (.....) To extract PC k-mers from protein sequences we first re-label each amino acid as one of seven groups based on its physio-chemical properties, as described by Shen et al. [30]: ({AGV}, {C}, {FILP}, {MSTY}, {HNQW}, {DE}, and {KR})."
